# Advances on genetic and genomic studies of ALV resistance

**DOI:** 10.1186/s40104-022-00769-1

**Published:** 2022-10-11

**Authors:** Guodong Mo, Ping Wei, Bowen Hu, Qinghua Nie, Xiquan Zhang

**Affiliations:** 1 Guangdong Provincial Key Laboratory of Agro-Animal Genomics and Molecular Breeding, College of Animal Science, South China Agricultural University, Guangzhou, 510642 Guangdong China; 2 Key Lab of Chicken Genetics, Breeding and Reproduction, Ministry of Agriculture, Guangzhou, 510642 Guangdong China; 3 State Key Laboratory for Conservation and Utilization of Subtropical Agro-Bioresources, South China Agricultural University, Guangzhou, 510642 Guangdong China; 4 Institute for Poultry Science and Health, Guangxi University, Nanning, 530001 Guangxi China

**Keywords:** Avian leukosis, Endogenous retrovirus, Gene editing, Immunity, Interferon-stimulated genes, Receptor, Resistant breeding

## Abstract

Avian leukosis (AL) is a general term for a variety of neoplastic diseases in avian caused by avian leukosis virus (ALV). No vaccine or drug is currently available for the disease. Therefore, the disease can result in severe economic losses in poultry flocks. Increasing the resistance of poultry to ALV may be one effective strategy. In this review, we provide an overview of the roles of genes associated with ALV infection in the poultry genome, including endogenous retroviruses, virus receptors, interferon-stimulated genes, and other immune-related genes. Furthermore, some methods and techniques that can improve ALV resistance in poultry are discussed. The objectives are willing to provide some valuable references for disease resistance breeding in poultry.

## Introduction

Avian leukosis (AL) is a general designation of a variety of avian neoplastic diseases caused by the avian leukosis virus (ALV) [1–3]. Base on the host range, the antigenic differences of viral envelope glycoproteins, virus interference experiments, and the molecular biological characteristics of the viral genome, the ALV family members are divided into 10 subgroups, from A to J [4]. A new subgroup K has also been found in local domestic chicken breeds [5]. The four subgroups F, G, H, and I, are derived from other bird species [4]. ALV can also be divided into exogenous viruses and endogenous viruses according to the biological characteristics of the retrovirus. They can spread vertically or horizontally, with vertical transmission being the main mode of transmission.

The seven subgroups A, B, C, D, E, J, and K are all derived from chickens, of which A, B, C, D, J, and K are exogenous viruses, while E is an endogenous virus [4]. Although ALV-E is an endogenous virus with low or no pathogenicity, it can interfere with detection of exogenous viruses and is inherited according to Mendelian laws [3, 6]. However, the subgroups A, B, C, D, and J have the ability to infect and cause disease in chickens [6]. Currently, the most prevalent ALV in China is subgroup J, followed by subgroup A and B, while subgroup C and D are seldom reported. In recent years, the host range of ALV-J infection has expanded [7, 8], from the earliest broilers [9], to laying hens [10], local chicken breeds [11, 12], and even mallards [13]. After infection with ALV, chickens grow stunted, production performance declines, immunosuppression, and the immune effect of the vaccines is reduced, which can seriously lead to death [14]. In addition, the slaughter efficiency is affected because of the apparent pathological changes in organs and tissues throughout the body. The economic losses from subclinical pathological effects following ALV infection may be greater than the clinical losses from neoplastic death, and this has resulted in huge economic losses to the poultry industry.

The *gp85* gene encodes the most variable structural protein in the ALV genome, which is associated with virus neutralization and viral host range [15, 16]. Based on this feature, ALV can mutate or recombine into new retrovirus strains [17–19]. In order to prevent and control the disease, scientists have developed vaccines, but the effect of vaccines cannot provide sufficient protection for chickens [20, 21]. There are vertical and horizontal transmission modes of ALV, and the commercial vaccines may also potentially result in ALV transmission [22, 23]. Only through rigorous eradication measures for generations, the ALV can be eliminated from the population. However, ALV still remains a major threat to the poultry industry. The eradication is not carried out on a national scale, especially in small-scale farms in China that commonly from the so-called Yellow-chicken local breeds, as eradication measures require financial and technical support.

Disease-resistant breeding of poultry may be an effective way to prevent and control ALV. After the domestication of chickens, egg production, growth rate, feather color, and other traits have been fully selected, and huge benefits have been generated. However, disease-resistant traits in poultry are progressing very slowly. The combination of traditional breeding methods, DNA molecular marker, gene editing, and genomic selection may be able to accelerate the progress of disease-resistant breeding. The purpose of this review is to provide an overview of progress in ALV disease-resistant breeding of poultry. In this review, we describe the progress of ALV disease resistance breeding in poultry from the endogenous retroviruses, virus receptor, interferon-stimulated genes (ISGs), and other immune-related genes. Moreover, we also attempt to highlight the practical methods for ALV disease-resistant breeding.

## Endogenous retroviruses can affect innate immunity

### The general features of chicken ERVs

Compared with exogenous retroviruses, endogenous retroviruses (ERVs) exist as stable genetic elements in the host genomes. ERVs are present in almost all vertebrates [24]; they make up about 3% of the chicken genome, which originates from exogenous retroviral infection of germline cells [25, 26]. ALV-E was the first chicken ERV identified in avian species, which exists at various segregating loci in the genome [27]. The gene sequence and structure of ERVs are very similar to those of exogenous retroviruses [28]. However, most ERVs lack the envelope protein domain owing to mutations (insertions, deletions, and substitutions) in genetic elements [29]. Usually, exogenous retroviruses are classified into seven genera, including Alpha-, Beta-, Gamma-, Delta-, Epsilon-, Lenti-, and Spuma-like, whereas ERVs do not follow the classification [30]. A total of 492 relatively complete ERVs have been detected with analysis of avian genomes using the RetroTector program [31]. Based on their relationships with exogenous retroviruses and the similarity and structural characteristics of polymerase gene, ERVs are roughly classified into three classes: class I ERVs are closely related to Gamma- and Epsilon-, class II ERVs are closely related to Beta-, and class III ERVs are closely related to Spuma-like [30–32]. Many ERVs cluster together in the promoters and introns, and have translational functions [32].

ERVs had long been considered as junk DNA, but now they have been identified as an important part of the body's immune mechanism [33, 34]. Although they are usually dormant, they can be reactivated by a variety of stimuli, including viral infection. For example, human immunodeficiency virus (HIV) can induce transcription and translation of ERVs viral elements [35, 36].

### The Effects of ERVs on the immune system

**Fig. 1:**
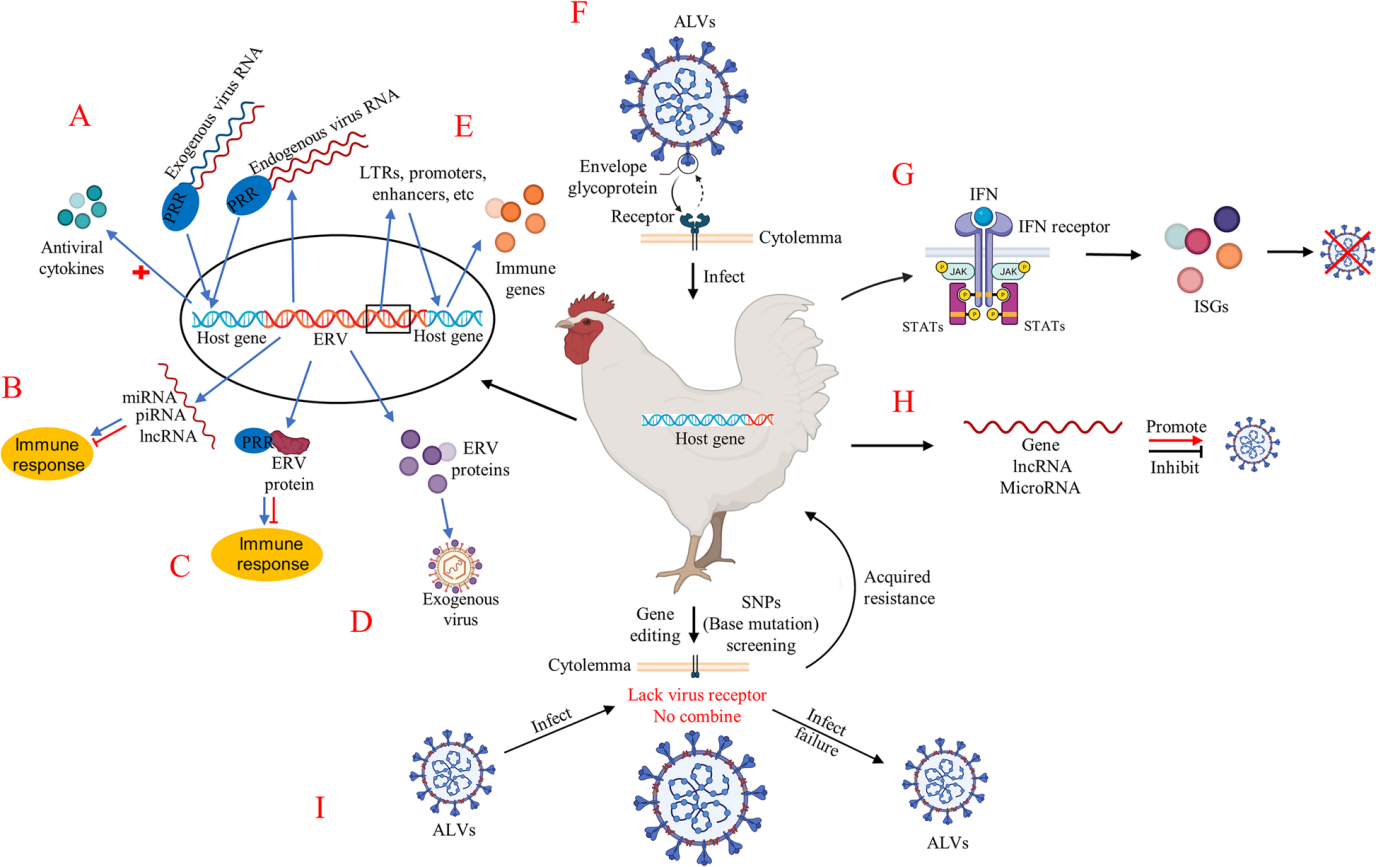
Host genes and genomes are closely related to avian leukosis virus (ALV) resistance. **A** Nucleic acids derived from ERVs can be considered as a type of pattern recognition receptors, which can recognize its complementary exogenous viral RNA and trigger a more specific immune response; **B** Small RNA molecules derived from ERVs, such as miRNA, piRNA, and lncRNA, can modulate the antiviral immune response in a direct manner; **C** ERVs-derived proteins modulate the antiviral immune response; **D** ERVs-derived proteins interfere with the receptors of exogenous viruses; **E** The retroviral elements of ERVs regulate host immune gene expression; **F** ALV invades the host by binding to the receptors on the host cell membrane; **G** IFNs bind their cognate receptors to induce ISGs through the JAK/STAT pathway after ALV infection; **H** Immune-related factors in the host genome can promote or inhibit virus replication after ALV infection; **I** Using gene editing methods to knock out the viral receptor gene or screen individuals with mutations in viral receptor gene, there is no viral receptor on the host cell membrane, ALV cannot bind to the receptor and infect the host, and the host will also acquire resistant to ALV

Replication competent ERVs can affect the host innate immune response and immune tolerance against exogenous retroviruses. Not all ERVs are genetically complete, and the expression of proteins encoded by a single gene can still have significant effects on the host [37–39]. ERVs have been identified that support antiviral immune responses through multiple mechanisms, such as enhancing cellular sensing, modulating viral gene expression, blocking viral binding to receptors, and limiting viral assembly (Fig. 1A-E) [40]. Nucleic acids, small RNA molecules (miRNA, piRNA, and lncRNA), and proteins derived from ERVs can sense or modulate immune responses (Fig. 1A-C). In addition, proteins derived from ERVs can inhibit viral infection by interfering with the receptors of exogenous viruses and preventing virus transport to cellular receptors (Fig. 1D). It is important to note that the retroviral elements of ERVs, such as promoters, enhancers, transcription start sites, and LTRs, may also contribute to regulating host immune gene expression (Fig. 1E).

### ERVs are a double-edged knife to chickens

ERVs have both positive and negative aspects in poultry. Insertion of ERVs into poultry genomes can result in the emergence of some desirable commercial traits, such as slow-feathering [41, 42], recessive white feathers [43], and green eggshell [44, 45]. In addition, the presence of ERVs can also affect the performance of chickens. ALV-E reduces growth rate and body weight in broilers [46, 47], as well as albumen height, egg weight, egg production, and sexual maturity in laying hens [48–50]. Not surprisingly, the immune effects of ERVs in poultry genes are contradictory. For example, a lncRNA (lnc-LTR5B) derived from ERV can regulating the cell surface translocation of BiP, which can be exploited by ALV-J to complete its life cycle [51]; while another lncRNA (lnc-ALVE1-AS1) can induce an antiviral innate immune response via a TLR3-dependent pathway in the cytoplasm [52].

In the poultry genome, if the host carries some ERVs (such as *ev21* or *ev6*), it is more susceptible to exogenous ALV-J than the non-carriers and more prone to tumors [53–55]. In addition, co-infection of ALV-E and serotype 2 Marek's disease virus (MDV-2) increases the incidence of lymphoid leukosis-like bursal lymphomas in susceptible chickens [56, 57]. Most seriously, ERVs can recombine with exogenous viruses to form new subgroups. ALV-J is thought to be evolve from EAV-HP and exogenous ALV recombination [58, 59]. We previously demonstrated that the ALV-J strain (M180) did not recombine with the ev21, indicating that a reorganization event needs to occur under specific conditions [39].

Fortunately, ERVs are not necessary for healthy chicken development. For the ERVs sites with commercial value, we can make use of them; and for the ERVs sites with negative effects, we can use the breeding strategy in line with their growth during the breeding process, or even we can eliminate them.

## ALV cannot invade host cells until it binds to viral receptors

Each virus has its own cell-surface receptors that interact with the virus to help it enter cells. These receptors are membrane proteins that have "normal" cellular functions, but can be hijacked by viruses to help them infect cells [60]. The relationship between viruses and receptors is not a simple one-to-one. Viruses can use several different proteins as their receptors, but sometimes a protein can also be used as a receptor by more than one virus [61, 62].

The ALV envelope glycoprotein contains surface glycoprotein (SU) encoded by *gp85* and transmembrane glycoprotein (TM) encoded by *gp37*. SU contains the main domain that interacts with receptors, and TM can anchor SU to the cell membrane (Fig. 1F) [63]. The amino acid (aa) sequences of envelope glycoproteins from ALV-A to ALV-E are highly conserved except for the five variable regions in SU (vr1, vr2, vr3, hr1, and hr2) [64]. Among these variable regions, the hr1 (aa194–198 and aa206–216) and hr2 (aa251–256 and aa269–280) regions bind to the receptor [65]. To date, all chicken breeds are susceptible to ALV-J. But quail and many other birds are resistant to ALV-J infection, suggesting that the viral receptor is species-specific in birds [66, 67]. However, ALV-A, B, C, and J do not share common host receptors, and they have their own specific receptor [68]. The receptors of each virus subgroup are introduced one by one below.

### The receptor for subgroup A and K avian leukosis virus

The subgroup A avian leukosis virus (ALV-A) enters the cell through the Tva receptor, which belongs to the family of low-density lipoprotein receptors (LDLR) [69]. The virus receptor of subgroup K is also Tva [70]. The *Tva* gene, encoding the receptor Tva, is located on chicken chromosome 28 and is orthologous to the mammalian gene, originally called *8D6A*. *8D6A* gene encodes a 282-aa protein (8D6 antigen) that is abundantly expressed on follicular dendritic cells, which contains two LDL-A modules and a transmembrane domain [71]. The viral interaction domain of Tva is determined by a 40-aa-long motif called the LDL-A module within the extracellular domain of Tva [72, 73]. This LDL-A module contains six cysteines and five acidic residues, which are highly conserved among all members of the LDL receptor superfamily [74]. The *Tva* homology between chicken and quail is approximately 65%, and most of the 11 aa differences are clustered at the N-terminus of the LDL-A module [71]. Although the physiological function of Tva remains at the speculative level, the cysteine residues of 2 and 3 at the N terminus of the Tva LDL-A module have a critical role in ALV-A entry [72].

*Tva* does not appear to be absolutely necessary for healthy chicken development, and other related signaling molecules may compensate for the loss of *Tva* [75]. Tva can be specifically recognized and bound by the envelope glycoproteins encoded by the envelope genes of ALV-A and ALV-K and promote their invasion [70]. The vr3 can affect the binding of ALV-A envelope glycoproteins to Tva, but the mutation (s7, s8, and K251E) of vr3 has no significant effect on ALV-K binding to Tva [65, 76]. Moreover, hr1 (aa 194–198 and aa 206–216) and hr2 (aa 251–256 and aa 269–280) of ALV-K played a key role in the binding to Tva. A single aa mutation (G196A and R198H) can abolish the binding of ALV-K to Tva [65]. There are six alleles (*Tva*^*r*^, *Tva*^*r2*^, *Tva*^*r3*^, *Tva*^*r4*^, *Tva*^*r5*^, and *Tva*^*r6*^), mostly owing to an intron 1 deletion (Table 1). These deletions disrupt mRNA splicing of the *Tva* receptor gene and prematurely introduced the TGA stop codon, thereby reducing sensitivity to ALV-A [77, 78]. Recent findings have demonstrated that ALV-A and ALV-K-resistant individuals can be produced after *Tva* knockout in chicken primordial germ cells (PGCs) using CRISPR/Cas9 gene editing technology [79].

**Table 1:** The base change sites and phenotypes of alleles of ALV receptors

Subgroup	Receptor	Alleles	Mutation mechanism	Base changes	Phenotype
ALV-A/K	Tva	*Tva*	-	-	Sensitive
		*Tva*^*r*^	Base mutation	C/G ^168 mRNA^	Resistant
		*Tva*^*r2*^	Exon 1 insert 4 bp	^284^CTGC^287^	Resistant
		*Tva*^*r3*^	Intron 1 miss 10 bp	^507^ACCCCGCCCC^516^	Reduce susceptibility
		*Tva*^*r4*^	Intron 1 miss 5 bp	^507^ACCCC^511^	Reduce susceptibility
		*Tva*^*r5*^	Intron 1 miss 10 bp	^502^CGCTCACCCC^511^	Reduce susceptibility
		*Tva*^*r6*^	Intron 1 miss 15 bp	^502^CGCTCACCCCGCCCC^516^	Reduce susceptibility
ALV-B/D/E	Tvb	*Tvb*^*s1*^	-	-	Sensitive to subgroups B/D/E
		*Tvb*^*s3*^	Base mutation	T/A ^184 mRNA^	Sensitive to subgroups B/D
		*Tvb*^*r*^	Base mutation	C/T ^172 mRNA^	Resistant
ALV-C	Tvc	*Tvc*	-	-	Sensitive
		*Tvc*^*r*^	Base mutation	C/A^165 mRNA^	Resistant
ALV-J	NHE1	*NHE1*	-	-	Sensitive
		*NHE1*^*∆*^	Base mutation	W38	Resistant

“-” means no change. The superscript numbers indicate gene loci

### The receptor for subgroup B, D, and E avian leukosis virus

The subgroups B, D, and E avian leukosis virus (ALV-B, D, E) share the same Tvb receptor, which belongs to the tumor necrosis factor receptor family (TNFR) [80–83]. The Tvb receptor contains three extracellular cysteine-rich domains (CRD1, CRD2, and CRD3) and a cytoplasmic death domain that can activate apoptosis in cells [80, 84]. Three *Tvb* alleles have been identified in chickens, in which the *Tvb*^*s1*^ allele encodes a TVB^S1^ receptor that is susceptible to these three subgroups, the *Tvb*^*s3*^ allele encodes a TVB^S3^ receptor that is only sensitive to subgroups B and D, and the *Tvb*^*r*^ allele encodes a receptor that is not infected by any subgroups (Table 1) [80, 83, 85].

**Fig. 2:**
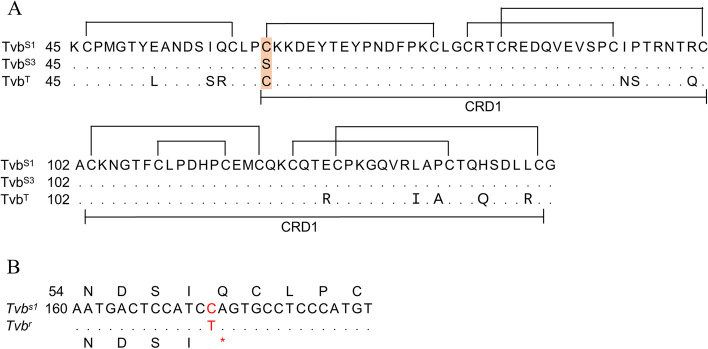
The mutational analysis of *Tvb*. **A** *Tvb*^*S1*^ and *Tvb*^*S3*^ differ by a serine-to-cysteine substitution at residue 62 (shaded) [83]. The regions of *Tvb*^*S1*^, *Tvb*^*S3*^, and *Tvb*^*T*^ that encompass amino acids 45 to 144, and the predicted intrachain disulfide bonds. **B** The *Tvb*^*r*^ allele contains a premature stop codon (indicated by an asterisk) [85]. Tvb^T^, turkey Tvb receptor

The main difference between TVB^S1^ and TVB^S3^ is residue 62 in the CRD2 domain, in which TVB^S1^ is Cys, while TVB^S3^ is Ser (Fig. 2A) [83, 85]. This mutation alters the structure of CRD2, resulting in host resistance to ALV-E. Furthermore, ALV-B and ALV-E have different disulfide bond requirements. Cys-46 and Cys-59 in TVB^S1^ form a disulfide bond, that is important for the receptor function of ALV-E [83, 86]. Residues Leu-36, Gln-37, and Tyr-42 in CRD1 of TVB^S1^ are essential for this receptor function [87], while the residues of Tyr-67, Asn-72, and Asp-73 in CRD2 of Tvb^S1^ are essential for efficient binding and entry of ALV-E (Fig. 2A) [88]. There is a nucleotide difference between the open reading frame (ORF) of *Tvb*^*s1*^ and *Tvb*^*r*^. Starting at residue 172 downstream of the methionine codon, *Tvb*^*s1*^ is cytosine and *Tvb*^*r*^ is thymidine (Fig. 2B) [85]. This mutation produces an in-frame stop codon (CAG → UAG), which encodes a severely truncated protein product that is resistant to ALV-B, ALV-D, and ALV-E infection [85].

The normal cellular function of chicken Tvb protein remains unclear, and its function can only be estimated based on its similarity to the TNFR protein family. The Tvb structure is similar to mammalian TNF-related DR4 and DR5 proteins [83]. Tvb receptor is a functional death receptor that can kill cells through the caspase pathway by a mechanism that is dependent upon the cytoplasmic death domains [80, 84]. Therefore, ALV-B infection can lead to the death of chicken embryo fibroblasts (CEFs) [80]. The frameshift mutation in Tvb may reduce the susceptibility to ALV [89]. Similarly, mutations in the virus transmembrane envelope glycoproteins may enhance host susceptibility to ALV [90].

### The receptor for subgroup C avian leukosis virus

The receptor of the subgroup C avian leukosis virus (ALV-C) is the Tvc receptor, which is similar to mammalian butyrophilins, members of the immunoglobulin superfamily [91]. Interestingly, both *Tvc* and *Tva* genes are closely linked on chromosome 28 [71, 92]. The Tvc cellular domain contains two immunoglobulin domains IgV and IgC, which both contain two conserved cysteine residues and a potential N-linked glycosylation site. Interacting with the glycoproteins of ALV-C is the IgV domain, in which there are at least two aromatic aa residues Trp-48 and Tyr-105 [92]. These aromatic aa residues are key determinants of receptor-virus interactions [93, 94]. IgV binds to ALV-C glycoprotein with low-nanomolar affinity [91, 93]. There are a certain number of individuals resistant to *Tvc* in chickens. In this situation, the *Tvc* gene is mutated, in which codon 55 (TGC, cysteine) is changed to a termination codon (TGA) (Table 1) [91]. Like Tva and Tvb, the normal cellular biological function of Tvc is unknown.

### The receptor for subgroup J avian leukosis virus

The receptor of the subgroup J avian leukosis virus (ALV-J) is the chicken Na ^+^ /H ^+^ exchanger 1 (*NHE1*) [95]. NHE is a transmembrane protein encoded by the *NHE* gene located on chromosome 23, which are named NHE1-NHE9 according to their order of discovery [95]. NHE1 is a housekeeping protein that regulates intracellular pH, Na ^+^—H ^+^ ion transport, and cell proliferation; it is expressed on almost all cell membranes [96]. Also, NHE1 is expressed in nearly all chicken breeds, which may be the reason why all chickens are susceptible to ALV-J [66, 67]. It was found that *NHE1* expression is significantly up-regulated in avian osteoclasts during differentiation [97]. During in vitro infection, *NHE1* expression was time-dependently upregulated. During in vivo infection, *NHE1* expression levels were closely related to tumor bearing and immune tolerance chickens, and NHE1 protein levels were increased in most tissues [98]. This means that the expression of mRNA and protein of NHE1 can be induced by ALV-J. NHE1 can regulate cell death, cell migration, proliferation, and survival through the MAPK signaling pathway [99]. The abnormal expression of NHE1 can cause intracellular pH imbalance, inhibit cell apoptosis, promote cell proliferation, and lead to tumorigenesis [100–102]. This may be the reason why chickens easily develop tumors in bone marrow, liver, and kidneys after infection with ALV-J.

The ALV-J binding site is in the non-conserved tryptophan at residue position 38 (W38) within the prominent first extracellular loop (ECL1) of NHE1 (Table 1) [103, 104]. The ECL1 domain of chicken NHE1 is highly conserved and has no aa polymorphisms [105]. In our previous research, we found 36 single nucleotide polymorphisms (SNPs) in the *NHE1* gene sequence, with an average of one SNP in every 170 bp [106]. Contrary to findings in chickens and turkeys, other poultry species (ducks, geese), and birds (Japanese quail, gray partridge, common pheasant, guinea fowl, and chukar) lack the crucial W38 [105]. Editing the *NHE1* gene is the first step in developing chickens resistant to ALV-J [107, 108].

There is not only one receptor for ALV-J, the chicken Annexin A2 (chANXA2) [109] and the chicken glucose-regulation protein 78 (chGRP78) [110] are also as novel receptors for ALV-J. However, their mechanism of action needs to be further studied. Furthermore, dPRLR in slow-feathering chickens may also be a receptor for ALV-J virus [111]. The susceptibility of the fast- and slow-feathering chickens to ALV-J is different, and the slow-feathering chickens are more susceptible to ALV-J [54, 112]. In the genome, slow-feathering chickens have two more genes than fast-feathering chickens: *ev21* and a fusion gene (*dSPEF2/dPRLR*) [113, 114]. The fusion gene is present in the genome of all slow-feathering chickens, but *ev21* is not [115]. Fusion genes are widely distributed in many tissues and cells, and have potential as viral receptors [116, 117].

## Host interferon-stimulated genes play an important role in ALV infection

### The general features of interferon-stimulated genes

In order to replicate and spread in the host, a virus must break through the powerful immune system. In turn, the host activates different cascades of signaling pathways participate in the immune response for resisting the invasion of the virus [118]. Activation of interferon (IFN) pathways is the most important events in the host–pathogen battle. IFNs play a critical role in the early immune response to viral infection, as IFNs bind to their cognate receptors and upregulate hundreds of interferon-stimulated genes (ISGs) through the JAK/STAT pathway (Fig. 1G). These genes interrupt viral replication and provide sufficient time for the development of an adaptive immune response [119, 120].

According to the homology and specificity receptors of IFNs, they are divided into type I (IFN-α, IFN-β), type II (IFN-γ), and type III (IFN-λ) [121]. The types I and III IFNs are considered to be the main antiviral cytokines, and type II IFNs also have antiviral properties [122]. Each IFNs can induce unique ISGs, but some ISGs are overlapping [123]. Furthermore, viral infection can induce ISGs production [124]. Some ISGs are not only capable of directed antiviral, but also enhance signaling from pattern recognition receptors in a synergistic manner, thereby enhancing the innate antiviral response [125].

### Many ISG genes with antiviral activity exist in poultry

In our previous study, we identified 205 type I ISGs, 299 type II ISGs and 421 type III ISGs in chickens [126]. A total of 152 potential ISGs were identified in the peripheral blood mononuclear cells of ALV-J-infected chickens [127]. In the process of ALV-J virus infection, the transcription levels of ISGs changed significantly, indicating that ISGs play an important role in ALV infection [12, 128, 129]. Subsequently, we studied the antiviral mechanism of two ISG genes (long-chain Acyl-CoA synthetase 1—*ACSL1*, cholesterol 25-hydroxylase—*CH25H*). *ACSL1* inhibits ALV-J virus replication by positively regulating the expression of IFN-I, and induces apoptosis through the PI3K/Akt signaling pathway [130]. *CH25H* inhibits ALV-J replication by producing 25-hydroxycholesterol [127].

The antiviral properties of many ISGs have been identified (Table 2). Some ISGs have been shown to have strong antiviral activity against ALV-J, but the antiviral mechanism of most ISGs remains unclear [127, 131]. IFN-induced ISGs have inhibitory effects on entire viral life cycles (entry, uncoating, transcription, translation, assembly, and egress) [119]. When two ISGs are expressed in combination, the efficacy of their antiviral activity is often greater than that of a single ISG [132]. During viral infection or IFN therapy, the expression levels, and species of ISGs usually depend on time, dose, and cell type [126, 132, 133]. Furthermore, taking advantage of these naturally existing ISGs may be an effective method in the development of novel drugs to treat AL [131].

**Table 2:** Antiviral interferon-stimulated genes

Gene	Targeted viruses	Viral life cycle	Mechanism related to antiviral activity	Reference
*ACSL1*	ALV-J	Replication	Regulating the expression of IFN-I	[130]
*CH25H*	ALV-J, PRRSV, RABV, reovirus, SARS-CoV-2	Replication	Producing 25-hydroxycholesterol, blocking membrane fusion,	[119, 127, 134–136]
*IFITM1/2/3*	HIV-1; IAVs, lyssaviruses; SARS-CoV	Entry; Restrict viral membrane hemifusion	Unknown, possibly target endocytic pathway	[119, 137–139]
*Mx*	ALV-J, IAVs, NDV, SeV, VSV	Unknown	Unknown	[119, 131, 140]
*OASL*	ALV-J, NDV, HCV	Unknown	Unknown	[119, 129, 141]
*PKR*	Capripoxvirus, PRRSV	Replication	Unknown	[119, 142, 143]
*ZC3HAV1* (*ZAP*)	NDV, PRRSV, Retrovirus	Post-entry, translation	Target viral RNA, promote RIG-I signaling	[119, 144–146]

*ALV-J* Subgroup J avian leukosis virus, *HCV* Hepatitis C virus, *IAVs* Influenza A viruses; *NDV* Newcastle disease virus, *PRRSV* Porcine reproductive and respiratory syndrome virus, *RABV* Rabies virus, *SARS-CoV* SARS coronavirus, *SeV* Sendai virus, *VSV* Vesicular stomatitis virus

## The immune response of poultry against ALV is very complex and there are other immune-related factors against and/or promoting ALV replication

Different genetic lines of chickens can affect viral infection/replication and utilize the mitochondrial respiration pathway differently [147, 148]. Due to intense selection for production traits, the immune cell metabolic capacity of the commercial lines is lower than that of the traditional lines [148]. Inbreeding also significantly affect the transcription of immune cell genes in their offspring [149]. In artificial breeding conditions, the genetic variability of coding elements of the chicken immune system is degenerated, which can decrease resistance to various diseases [150]. In the host, ALV has two states, persistent viremia, and intermittent viremia [151, 152].

The genes on chromosome 16 of chickens are all immune-related genes, and there are still many immune-related genes on other chromosomes [153]. To better understand the mechanism of ALV replication and host pathogenesis, multiple research teams performed transcriptome sequencing analysis of different types of cells (CEF, HD11, MDM), and organs (liver, spleen), including DNA methylation, m^6^A RNA methylation, mRNA, miRNA, lncRNA, and circRNA [152–157]. Even single-cell sequencing has been used to understand the development of lymphocytes in the host after infection with ALV and the response to the virus [158]. In these results, a large number of differentially expressed genes were found in each RNA-seq, only a few of which we have some understanding. Based on genome sequencing, we can fully understand the mechanism of action of the host’s immune system after infection with the virus, find the gene positions of genetic variation that can improve disease resistance in poultry, and then use these gene loci for selective breeding.

Among the sequencing results, some of these genes can promote the host immune response to inhibit viral replication [52, 130], while others inhibit the host immune response to promote viral replication (Fig. 1H) [159–161]. For those genes with antiviral functions, we do not need to worry too much, just screen out it from the genome and understand its mechanism of action. More attention should be paid to those genes that help the virus to survive and replicate in the host, because through long-term interactions with the host, viruses have evolved various mechanisms to fight and evade the host’s antiviral response [162–164]. In addition, we need to pay attention to the SNP loci in the gene. Studies have shown that SNP loci can significantly affect the apparent traits of animals [165]. Genotypes of chickens that are resistant to ALV can be found by association analysis of SNPs in genomes [106]. Notably, hormones are also a non-negligible factor in viral infections, which play an important role in viremia, viral replication, and the host immune response [111, 129, 166]. In poultry breeding, hormone content and SNP typing of immune-related genes may be a direction for selection.

## Strategies and techniques of disease-resistant breeding for ALV

Although ALV has been studied for decades, there are still many gaps in scientific knowledge, including tumor causing, immune suppression, and immune escape [151]. It is noteworthy that the oncogenic mechanisms of ALV and MDV are different. MDV carries an oncogene that it can directly induce tumor formation in the body, whereas ALV are integrated with specific cellular genes by its proviral DNA, and the insertion of the viral promoter adjacent to this gene results in its enhanced expression, leading to neoplasia [167]. Due to the rapid evolution of ALV, there are still no effective treatments and vaccines available, and other biosecurity measures may also be insufficient. Therefore, traditional breeding methods can be used to develop disease resistance or tolerance.

ERVs might be considered as a potential genetic selection [168]. More than 400 ERVs have been identified in the poultry genome; and we know about a few of them, and the rest remain unknown [131]. ERVs in the host have both positive and negative aspects. The random insertion of ERVs into genes can produce some traits of commercial value, but their insertion can also make the host more susceptible to exogenous viruses and increase the chance of recombination of exogenous viruses. For example, the insertion of *ev21* into the genome led to the emergence of a commercial trait of great utility in chickens, the slow-feathering plumage trait, but the susceptibility of the host to ALV-J was significantly increased [53, 54, 111]. However, the growth and development of fast-feathering chickens without *ev21* gene was no different from that of other individuals, indicating that *ev21* is not an essential gene for the host. In fact, we can eliminate individuals with associated ERVs from the genome as needed. If we want to take advantage of these special ERVs, we need to develop more stringent and effective biosecurity measures, and more complete disease purification measures.

Viruses only enters the target cells after binding to the host cell receptor protein [169]. The integration of proviral DNA of ALV is a random and unforeseen event. This demonstrates the importance of preventing or interfering with the binding of viruses to receptors on cells, and it also suggests that viral infections can be avoided by modifying/deleting host cell receptors. Fortunately, the receptors associated with several ALV subgroups in poultry are known, including their genetic structure and the amino acid sites to which the virus binds, as well as alleles for resistance to ALV (Table 1). Chinese local chicken breeds have high genetic selection potential owing to the high frequency of *Tva* and *Tvb* resistance alleles in their genomes [170]. Resistance of the subgroup C will be considered later, because it is rarely found in domestic chicken flocks. Improving resistance to the subgroup J is currently the most urgent. The gene sequence of *NHE1* in domestic chicken breeds is very conserved, which may be the reason why the subgroup J is popular in China [171]. This may serve as a drug target or a key gene locus for disease resistance breeding selection.

Through transcriptome sequencing technology, we can mine more ISG genes and immune-related genes that can improve disease resistance of poultry from the poultry genome. We can pinpoint these genes and use their genotypes (SNPs) for selective breeding. Gene editing can also be coordinated through selective breeding, because it precisely edits target loci identified in genome sequencing data, and introduces the new alleles associated with important economic traits. A combination of genomics and gene editing technologies will speed up the breeding of poultry for disease resistance [172, 173]. In the past few decades, different gene editing technologies have been established, and a combination of the PGC-mediated method and CRISPR/Cas9 system is the most widely used gene editing method [172–174].

Koslová et al. [108] used CRISPR/Cas9 genome editing tools to introduce frame-shifting indel mutations into *Tva*, *Tvc*, and *NHE1* loci encoding receptors for the ALV subgroups A, C, and J, respectively. For all three loci, the homozygous frame-shifting indels generating premature stop codons induced phenotypes which were fully resistant to the virus of respective subgroup in DF-1 cells. Excitingly, chicken PGC were edited by CRISPR/Cas9 gene editing technology and a genetically engineered commercial chicken line (NHE1 ΔW38 chickens) resistant to ALV-J was successfully established [175–177]. In other words, the mutation NHE1 ΔW38 mediates the chicken resistance against HPRS-103, and the W38 deletion has no negative effects on chicken growth and health [175–177]. Furthermore, using the same method, a transgenic commercial chicken line resistant to ALV-A/K was obtained [79]. Those results shows that the viral receptor of ALV is not necessary in the growth cycle of chickens, and it can be eliminated to obtain resistance (Fig. 1I). If the binding receptor sites of different subpopulations are edited simultaneously, it is possible to confer resistance to multiple ALV subgroups in poultry. Moreover, CRISPR/Cas9 gene editing technology may reduce the time and cost of breeding poultry for disease resistance.

## Conclusions

Compared with the growth, egg production, meat production and other commercial traits of poultry, the progress of breeding for disease resistance in poultry has been slow. Despite our knowledge of the viral properties of AL, and the immune response of poultry, there are still significant gaps in our understanding of the antiviral immune response in the poultry genome. In this review, we summarize the recent progress in research regarding on the genes related to ALV infection in the poultry genome. This information could further promote the research regarding breeding for poultry disease resistance.

In general, according to the above-mentioned research results, it is possible to obtain ALV resistant varieties by combining traditional breeding methods, genomic selection technology and gene editing technology.

## Data Availability

Not applicable.

## Abbreviations

aa: Amino acid
AL: Avian leukosis
ALV: Avian leukosis virus
ALV-A/B/C/D/E/J: Subgroups A/B/C/D/E/J avian leukosis virus
CRD: Cysteine-rich domains
ECL: Extracellular loop
ERVs/ev: Endogenous retroviruses
HIV: Human immunodeficiency virus
ISGs: Interferon-stimulated genes
LDL: Low-density lipoprotein
LTRs: Long terminal repeats
NHE: Na ^+^ /H ^+^ exchanger
PGC: Primordial germ cells
SNPs: Single nucleotide polymorphisms
SU: Surface glycoprotein
TM: Transmembrane glycoprotein
TNFR: Tumor necrosis factor receptor family
